# Clinical, laboratory, operative, and appendiceal morphometric correlates of histopathological appendicitis severity: A retrospective cohort study from Palestine

**DOI:** 10.1371/journal.pone.0357811

**Published:** 2026-09-09

**Authors:** Ali Shakhshir, Mahdi Aljamal, Abdallah Alem, Ibrahim Aljamal, Zainah Amjad Issa

**Affiliations:** 1 Department of Clinical Medicine, Faculty of Medicine, Arab American University, Jenin, Palestine; 2 Scientific Research Committee, Faculty of Medicine, Arab American University, Jenin, Palestine; 3 Department of Surgery, Faculty of Medicine, Arab American University, Jenin, Palestine; 4 Department of Surgery, Al-Makassed Islamic Charitable Society Hospital, Jerusalem, Palestine; 5 Faculty of Medicine, Arab American University, Jenin, Palestine; Jinnah Sindh Medical University, National Institute of Child Health, PAKISTAN

## Abstract

**Background:**

Clinical, laboratory, and imaging correlates of complicated appendicitis are well studied, but routinely recorded gross-pathology morphometrics and pathology-confirmed evidence from Palestine are less well characterized. We evaluated correlates of histopathological severity and adjusted associations with complicated appendicitis.

**Methods:**

This retrospective cohort included appendectomy encounters at Al-Makassed Islamic Charitable Society Hospital from December 2015 to July 2025. Source reconciliation identified 1,287 unique encounters; 245 histologically normal appendices and 32 records without a classifiable severity category were excluded, leaving 1,010 patients. Complicated appendicitis was defined as gangrenous or perforated disease. Group comparisons used chi-square or Monte Carlo exact tests and Kruskal-Wallis tests. A prespecified logistic model included age, sex, appendicolith, white blood cell count (WBC), and C-reactive protein (CRP).

**Results:**

The cohort included 227 catarrhal/simple, 458 suppurative, 262 gangrenous, and 63 perforated cases. Operative approach, nausea and/or vomiting, other intraoperative findings, appendicolith, WBC, neutrophil percentage, operative duration, and hospital stay differed across severity categories. In the adjusted model (n = 991), appendicolith was associated with higher odds of complicated appendicitis (adjusted odds ratio 2.20, 95% confidence interval 1.37–3.52; P = 0.001), whereas WBC showed an unexpected inverse association (adjusted odds ratio 0.938 per 1 × 10⁹/L, 95% confidence interval 0.906–0.971; P < 0.001). Age, sex, and CRP were not independently associated. Discrimination was weak (area under the receiver operating characteristic curve 0.594, 95% confidence interval 0.557–0.632). Maximum diameter, wall thickness, and appendix length showed no significant adjusted linear associations.

**Conclusions:**

Appendicolith was associated with complicated appendicitis, but weak discrimination and limited explained variation preclude use of the model as a clinical prediction tool. The inverse WBC association and negative linear morphometric findings require cautious interpretation.

## Introduction

Acute appendicitis is among the most common surgical emergencies. Distinguishing uncomplicated from complicated disease is clinically relevant because severity influences the suitability of non-operative management, operative complexity, antimicrobial treatment, postoperative care, and prognosis [1,2]. However, the biological and clinical boundary between uncomplicated and complicated appendicitis is imperfect, and no single symptom, laboratory marker, or imaging feature reliably classifies severity in all patients [3,4].

Most contemporary differentiation strategies combine clinical features, inflammatory biomarkers, and imaging findings. Computed-tomography features can improve classification, and several multivariable scores have been developed or validated for this purpose [5–11]. Nevertheless, performance varies across settings, and many models depend on variables that are not uniformly available in retrospective hospital records. Routine inflammatory markers such as white blood cell count (WBC) and C-reactive protein (CRP) are frequently studied, but their independent contributions are inconsistent across cohorts [3,12].

Prior studies have evaluated clinical, laboratory, imaging, and anatomical correlates of appendicitis severity, but routinely recorded gross-pathology morphometrics have not been assessed consistently, and pathology-confirmed evidence from Palestine remains limited [13–16]. We therefore aimed to characterize differences across four histopathological severity categories and to estimate adjusted associations with complicated appendicitis using routinely available clinical, laboratory, operative, postoperative, and gross-pathology variables. The regression was designed to estimate associations rather than to develop a clinical prediction rule.

## Materials and methods

### Study design, setting, and reporting

This retrospective cohort study was conducted at Al-Makassed Islamic Charitable Society Hospital in Jerusalem, Palestine, and included appendectomy encounters between December 2015 and July 2025. The study is reported in accordance with the Strengthening the Reporting of Observational Studies in Epidemiology (STROBE) statement [17]. The source cohort included pediatric and adult patients treated within routine clinical practice.

### Participants and cohort reconstruction

All appendectomy records identified during the study period were screened. The clinical source contained 1,287 unique Patient ID-surgery date encounters. Patients were eligible for the severity cohort when appendectomy had been performed and histopathological examination classified the appendix as catarrhal/simple, suppurative, gangrenous, or perforated appendicitis. We excluded 245 encounters in which histopathology showed a normal appendix and 32 encounters without a classifiable histopathological severity category. The final cohort therefore comprised 1,010 unique appendectomy encounters. Because all eligible records were included, no a priori sample-size calculation was performed.

Before reanalysis, the ID-linked clinical and morphometric source workbooks were reconciled using Patient ID and date of surgery. Duplicate encounter rows were consolidated so that the analytic data set contained one row per unique encounter. The clinical source was used for shared demographic, laboratory, operative, and postoperative variables; the morphometric source was used only for maximum appendiceal diameter, wall thickness, and appendix length after linkage and duplicate consolidation. When one duplicate morphometric record was missing and the other contained a value, the available value was retained. Distinct conflicting nonmissing values that could not be verified were treated as missing rather than resolved by assumption.

### Data sources and study variables

Data were abstracted from routinely maintained medical, operative, and pathology records using a structured data-collection sheet. Variables included age; sex; nausea and/or vomiting; CRP; WBC; neutrophil percentage; operative approach; operative duration; other intraoperative findings; appendicolith; hospital length of stay; wound infection; postoperative intra-abdominal abscess; and the three appendiceal morphometric measurements. Appendicolith status was derived from available operative and/or pathology documentation. Detailed preoperative imaging findings, symptom duration, preoperative antibiotic exposure, American Society of Anesthesiologists class, diabetes, immunosuppression, and other comorbidity measures were not consistently available and were not included in the adjusted model.

### Histopathological severity and binary outcome

The primary descriptive outcome was the histopathological severity category: catarrhal/simple, suppurative, gangrenous, or perforated appendicitis. For multivariable analysis, uncomplicated appendicitis comprised catarrhal/simple and suppurative disease, whereas complicated appendicitis comprised gangrenous and perforated disease. This binary definition was prespecified for the reconstructed analysis and was derived directly from the histopathological category rather than from the inconsistent legacy binary field.

### Gross-pathology morphometric variables

Maximum appendiceal diameter, appendiceal wall thickness, and appendix length were abstracted in centimeters from the gross-pathology information available in routine pathology records. These were clinical record measurements rather than prospectively standardized research measurements. The available records did not consistently document whether each measurement was obtained before or after fixation, the measuring instrument, handling of fragmented specimens, blinding to clinical information, or interobserver reliability. Imaging-derived morphometrics were not systematically available and could not be compared with the gross-specimen measurements. These limitations were considered when interpreting morphometric results.

### Data quality and missing-data handling

Raw source values were preserved in the audit file. Five WBC values below 1.0 × 10⁹/L and six neutrophil values below 1% were treated as missing in the cleaned analysis variables because they were incompatible with the stated units and could not be verified. One isolated wall-thickness value of 3.0 cm was also treated as missing because it was implausible relative to the remaining recorded range and could not be verified; it was not changed to an assumed replacement value. No statistical imputation was performed. Descriptive and group-comparison analyses used available observations for each variable. Accordingly, each four-category comparison was performed separately using all nonmissing observations for that specific variable; patients were not excluded from other analyses solely because a different variable was missing. Regression models used complete cases for the variables included in the respective model. Variable-specific availability and missingness are reported in S1 Appendix.

### Statistical analysis

Categorical variables are summarized as counts and percentages based on available observations. Continuous variables are summarized as medians and interquartile ranges because several distributions were non-normal and contained ties or outlying values. Comparisons across the four histopathological categories used Pearson chi-square tests when expected-cell assumptions were adequate. For sparse 2 × 4 tables, Fisher-Freeman-Halton exact tests with Monte Carlo estimation (100,000 samples; 99% Monte Carlo confidence interval) were used. Cramer’s V was calculated as an effect-size measure. Continuous variables were compared using Kruskal-Wallis tests. For variables with a significant omnibus result, pairwise comparisons used Mann-Whitney U tests with Bonferroni adjustment.

The primary multivariable binary logistic regression included age, sex, appendicolith, WBC, and CRP entered simultaneously. These covariates were selected a priori because of clinical relevance, availability, and prior literature; automated stepwise selection was not used. Operative approach, operative duration, and hospital stay were not included because they may reflect treatment selection or consequences of disease severity rather than baseline factors. Continuous predictors were retained in their original units. Linearity in the logit was examined with Box-Tidwell interaction terms, using a Bonferroni-adjusted threshold of 0.0167 for three tests. Multicollinearity was assessed with tolerance and variance inflation factors. Model performance was summarized using the likelihood-ratio chi-square test, Cox-Snell and Nagelkerke R2, the Hosmer-Lemeshow test, the area under the receiver operating characteristic curve (AUC) with 95% confidence interval, and the Brier score. Nonparametric bootstrap internal validation used 300 resamples to estimate optimism-corrected AUC and mean calibration intercept and slope. A sensitivity model expressed CRP on the log2 scale, interpreted per doubling.

Each morphometric variable was first examined in an unadjusted logistic model and then in a separate model adjusted for the five core covariates. Maximum diameter and wall thickness were scaled per 0.1 cm and appendix length per 1 cm. Because the Box-Tidwell assessment suggested possible curvature for appendix length, exploratory sensitivity analyses added a centered quadratic appendix-length term. A combined complete-case model included all three morphometric variables and the quadratic term; case-influence diagnostics and a high-leverage sensitivity analysis were used to assess robustness. These nonlinear analyses were exploratory and were not used to redefine the primary conclusion. All tests were two-sided, with P < 0.05 considered statistically significant unless a multiplicity-adjusted threshold was specified. Main analyses were conducted in IBM SPSS Statistics version 22 (IBM Corp., Armonk, NY, USA); the source audit and bootstrap calculations were performed in Python 3.11.

### Ethics statement

The study was approved by the Institutional Review Board of Arab American University, Palestine (IRB reference J-2025/A/2026/N) on 8 July 2025. The Arab American University Institutional Review Board provided ethical oversight for the research, and institutional permission to access the records was obtained from Al-Makassed Islamic Charitable Society Hospital, the data-holding clinical site. The study was conducted in accordance with the Declaration of Helsinki. The Institutional Review Board waived the requirement for individual informed consent because the study was a retrospective review of routinely collected, de-identified medical and pathology records with no direct patient contact or intervention. Data were accessed only by authorized study personnel and were handled in de-identified form for analysis.

## Results

### Cohort reconstruction and participant flow

**Fig 1:**
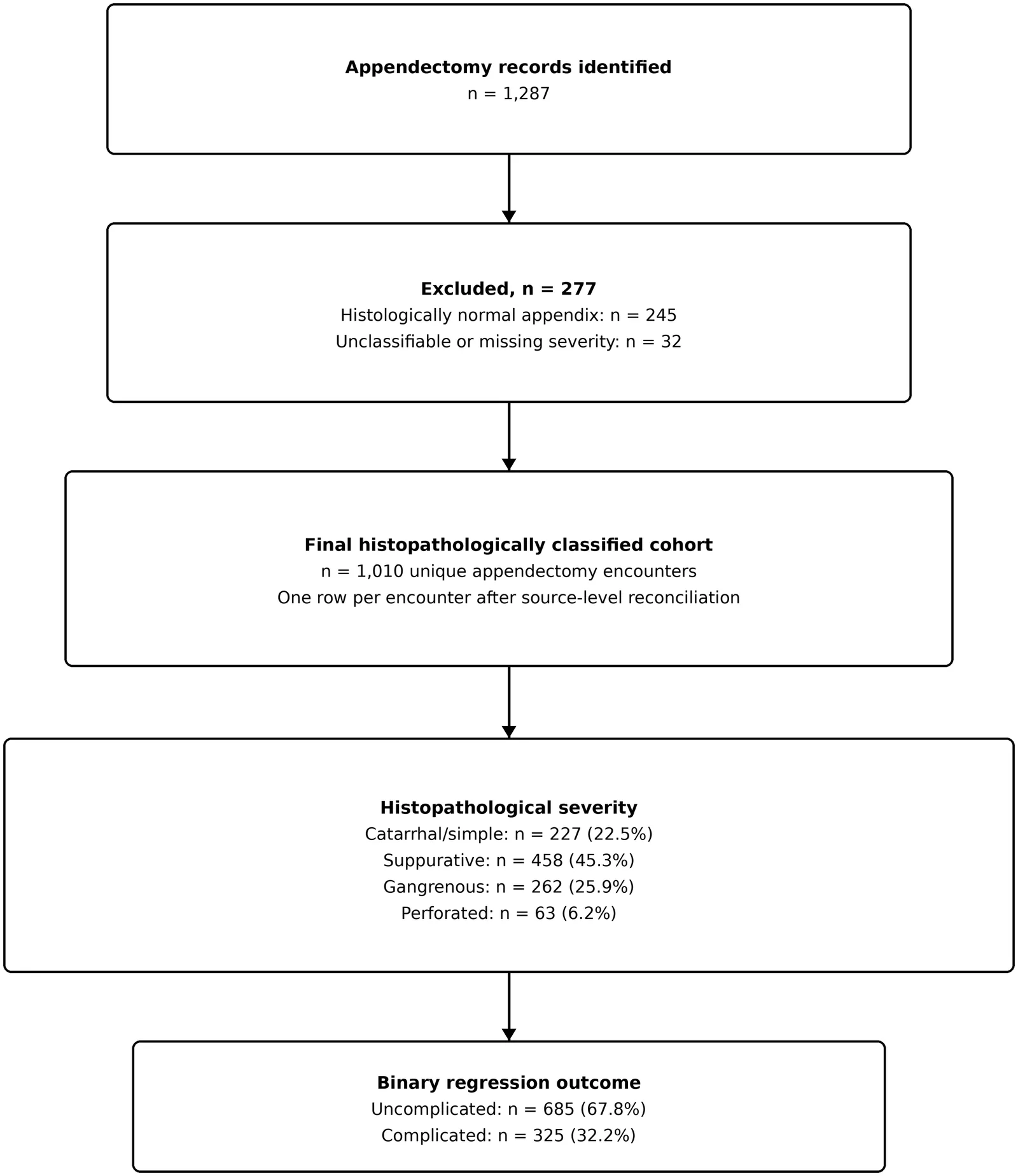
Participant selection and cohort reconstruction. The final cohort contained one row per unique Patient ID-surgery date encounter after source-level reconciliation.

A total of 1,287 unique appendectomy encounters were identified. Histopathology showed a normal appendix in 245 encounters, and 32 additional encounters did not have a classifiable histopathological severity category. The final analytic cohort therefore comprised 1,010 unique encounters (Fig 1).

### Overall cohort characteristics

Available observations differed by variable because of incomplete routine documentation. The available N is displayed directly beside every variable in Tables 1–4 and complete missingness information is provided in S1 Appendix. The cohort included 499 male and 510 female patients among 1,009 records with documented sex. Laparoscopic appendectomy was performed in 888/1,010 encounters (87.9%). Appendicolith was documented in 80/1,004 encounters (8.0%). Wound infection and postoperative abscess were uncommon, occurring in 18/1,010 (1.8%) and 7/1,010 (0.7%), respectively (Table 1). The median age was 40.5 years (interquartile range [IQR], 28.0–54.0), and the median hospital stay was 2 days (IQR, 2–3). Morphometric availability varied, with wall thickness available in 695/1,010 encounters (68.8%) (Table 2; S1 Appendix).

**Table 1:** Overall demographic, clinical, operative, histopathological, and postoperative characteristics.

Variable	Category	n (%)
Sex (N = 1,009)	Male	499 (49.5)
	Female	510 (50.5)
Operative approach (N = 1,010)	Open	122 (12.1)
	Laparoscopic	888 (87.9)
Nausea and/or vomiting (N = 940)	No	793 (84.4)
	Yes	147 (15.6)
Other intraoperative finding (N = 1,006)	No	965 (95.9)
	Yes	41 (4.1)
Appendicolith (N = 1,004)	Absent	924 (92.0)
	Present	80 (8.0)
Histopathological severity (N = 1,010)	Catarrhal/simple	227 (22.5)
	Suppurative	458 (45.3)
	Gangrenous	262 (25.9)
	Perforated	63 (6.2)
Binary severity (N = 1,010)	Uncomplicated	685 (67.8)
	Complicated	325 (32.2)
Wound infection (N = 1,010)	No	992 (98.2)
	Yes	18 (1.8)
Postoperative abscess (N = 1,010)	No	1,003 (99.3)
	Yes	7 (0.7)

Percentages use available observations as the denominator. Uncomplicated appendicitis includes catarrhal/simple and suppurative disease; complicated appendicitis includes gangrenous and perforated disease.

**Table 2:** Overall continuous characteristics and morphometric measurements.

Variable	Median (IQR)
Age, years (N = 1,010)	40.5 (28.0-54.0)
CRP, mg/L (N = 998)	12.7 (8.5-17.5)
WBC, × 10⁹/L (N = 1,002)	10.7 (7.7-13.8)
Neutrophils, % (N = 1,000)	60.7 (51.5-70.5)
Hospital stay, days (N = 1,009)	2 (2-3)
Operative duration, min (N = 900)	45 (35-50)
Maximum appendiceal diameter, cm (N = 903)	0.5 (0.5-0.5)
Appendiceal wall thickness, cm (N = 695)	0.3 (0.3-0.5)
Appendix length, cm (N = 904)	6.0 (4.5-7.5)

IQR, interquartile range; CRP, C-reactive protein; WBC, white blood cell count. Values are based on available observations.

**Table 3:** Categorical characteristics across histopathological severity categories.

Variable	Category	Catarrhal/simple (total n = 227)	Suppurative (total n = 458)	Gangrenous (total n = 262)	Perforated (total n = 63)	P value
Sex (N = 1,009)	Male	123 (54.2)	226 (49.5)	120 (45.8)	30 (47.6)	0.319
	Female	104 (45.8)	231 (50.5)	142 (54.2)	33 (52.4)	
Operative approach (N = 1,010)	Open	13 (5.7)	61 (13.3)	33 (12.6)	15 (23.8)	0.001
	Laparoscopic	214 (94.3)	397 (86.7)	229 (87.4)	48 (76.2)	
Nausea and/or vomiting (N = 940)	No	183 (90.1)	351 (82.0)	213 (86.6)	46 (73.0)	0.003
	Yes	20 (9.9)	77 (18.0)	33 (13.4)	17 (27.0)	
Other intraoperative finding (N = 1,006)	No	221 (97.4)	444 (97.6)	249 (95.0)	51 (82.3)	<0.001
	Yes	6 (2.6)	11 (2.4)	13 (5.0)	11 (17.7)	
Appendicolith (N = 1,004)	Absent	214 (94.3)	426 (93.8)	243 (93.1)	41 (66.1)	<0.001
	Present	13 (5.7)	28 (6.2)	18 (6.9)	21 (33.9)	
Wound infection (N = 1,010)	No	223 (98.2)	454 (99.1)	255 (97.3)	60 (95.2)	0.067
	Yes	4 (1.8)	4 (0.9)	7 (2.7)	3 (4.8)	
Postoperative abscess (N = 1,010)	No	226 (99.6)	454 (99.1)	260 (99.2)	63 (100.0)	1.000
	Yes	1 (0.4)	4 (0.9)	2 (0.8)	0 (0.0)	

Data are n (column %). Total group sizes are shown in the column headings, and the available overall N is shown beside each variable. For variables with missing data, percentages were calculated using the nonmissing observations within the corresponding histopathological category; the sum of counts for each variable therefore indicates the analyzed denominator. Pearson chi-square tests were used for sex, operative approach, and nausea and/or vomiting. Fisher-Freeman-Halton exact tests with Monte Carlo estimation (100,000 samples; 99% Monte Carlo confidence interval) were used for other intraoperative findings, appendicolith, wound infection, and postoperative abscess. No values were imputed.

**Table 4:** Continuous characteristics across histopathological severity categories.

Variable	Catarrhal/simple(total n = 227)	Suppurative(total n = 458)	Gangrenous(total n = 262)	Perforated(total n = 63)	P value
Age, years (N = 1,010)	42 (28-56)	39 (27-53)	39 (28-53)	46 (33-55)	0.110
CRP, mg/L (N = 998)	12.5 (8.5-15.8)	13.3 (8.5-18.7)	12.6 (8.5-16.9)	11.9 (8.5-19.7)	0.079
WBC, × 10⁹/L (N = 1,002)	10.7 (7.6-13.8)	11.4 (8.5-14.5)	9.8 (6.7-13.3)	10.7 (6.9-13.6)	0.004
Neutrophils, % (N = 1,000)	60.6 (51.9-71.5)	61.5 (52.0-70.5)	58.5 (49.5-68.5)	64.3 (53.3-76.7)	0.021
Hospital stay, days (N = 1,009)	2 (2−2)	2 (2-3)	2 (2-3)	2 (2-3)	0.042
Operative duration, min (N = 900)	45 (35-50)	45 (35-50)	45 (35-50)	50 (40-60)	0.001
Maximum diameter, cm (N = 903)	0.5 (0.5-0.5)	0.5 (0.5-0.5)	0.5 (0.5-0.5)	0.5 (0.5-0.5)	0.806
Wall thickness, cm (N = 695)	0.3 (0.3-0.5)	0.3 (0.3-0.5)	0.3 (0.3-0.5)	0.3 (0.3-0.5)	0.070
Appendix length, cm (N = 904)	6.0 (4.5-7.5)	6.0 (4.5-7.2)	5.5 (4.0-7.5)	5.0 (4.2-6.8)	0.273

Data are median (IQR) based on available observations for each variable. Total group sizes are shown in the column headings, and the available overall N is shown beside each variable. P values are from Kruskal-Wallis tests. No values were imputed. Adjusted pairwise comparisons for significant omnibus tests are reported in S1 Appendix.

### Categorical characteristics across severity categories

Histopathological severity was catarrhal/simple in 227 patients (22.5%), suppurative in 458 (45.3%), gangrenous in 262 (25.9%), and perforated in 63 (6.2%). Thus, 685 patients (67.8%) had uncomplicated and 325 (32.2%) had complicated appendicitis. Operative approach (P = 0.001), nausea and/or vomiting (P = 0.003), other intraoperative findings (P < 0.001), and appendicolith (P < 0.001) differed across severity categories. Appendicolith was present in 33.9% of perforated cases compared with 5.7%−6.9% in the other categories. Sex, wound infection, and postoperative abscess did not differ significantly across severity categories (Table 3). Cramer’s V was largest for appendicolith (0.246), followed by other intraoperative findings (0.185).

### Continuous characteristics across severity categories

WBC (P = 0.004), neutrophil percentage (P = 0.021), operative duration (P = 0.001), and hospital stay (P = 0.042) differed across the four categories, whereas age, CRP, maximum diameter, wall thickness, and appendix length did not show significant omnibus differences (Table 4). After Bonferroni adjustment, WBC was lower in gangrenous than suppurative appendicitis (adjusted P = 0.002), neutrophil percentage was higher in perforated than gangrenous appendicitis (adjusted P = 0.040), and operative duration was longer in perforated appendicitis than in each of the other three categories (all adjusted P ≤ 0.008). No pairwise hospital-stay comparison remained significant after adjustment (S1 Appendix).

### Primary adjusted association model

The primary logistic regression included 991 complete cases, of whom 320 had complicated appendicitis. The overall model was statistically significant (likelihood-ratio chi-square = 26.895, 5 degrees of freedom, P < 0.001) but explained little outcome variation (Nagelkerke R2 = 0.037). Appendicolith was positively associated with complicated appendicitis (adjusted odds ratio [aOR], 2.198; 95% confidence interval [CI], 1.374–3.516; P = 0.001). WBC showed an inverse association (aOR per 1 × 10⁹/L, 0.938; 95% CI, 0.906–0.971; P < 0.001). Age, male sex, and CRP were not independently associated (Table 5).

**Table 5:** Primary multivariable logistic regression for complicated appendicitis.

Variable	B	SE	Adjusted OR	95% CI	P value
Appendicolith present (yes vs no)	0.788	0.240	2.198	1.374-3.516	0.001
WBC, per 1 × 10⁹/L	−0.064	0.018	0.938	0.906-0.971	<0.001
CRP, per 1 mg/L	0.006	0.004	1.006	0.998-1.013	0.160
Age, per year	0.001	0.004	1.001	0.992-1.009	0.899
Male sex (vs female)	−0.188	0.138	0.828	0.632-1.086	0.173

Analytic n = 991, including 320 complicated events. All predictors were entered simultaneously. OR, odds ratio; CI, confidence interval; SE, standard error; CRP, C-reactive protein; WBC, white blood cell count. Model likelihood-ratio chi-square = 26.895 (df = 5), P < 0.001; Nagelkerke R2 = 0.037; Hosmer-Lemeshow P = 0.787; AUC = 0.594 (95% CI, 0.557–0.632).

### Model performance, assumptions, and sensitivity analyses

No meaningful multicollinearity was identified (variance inflation factor range, 1.004–1.035). Box-Tidwell interaction terms did not cross the Bonferroni-adjusted threshold for nonlinearity. The Hosmer-Lemeshow test did not indicate lack of fit (chi-square = 4.717, 8 degrees of freedom, P = 0.787), but discrimination was weak (AUC, 0.594; 95% CI, 0.557–0.632). The bootstrap optimism-corrected AUC was 0.582; the bootstrap mean calibration intercept and slope were −0.078 and 0.891, respectively. The Brier score was 0.213. Findings were materially unchanged when CRP was modeled per doubling: appendicolith aOR 2.221 (95% CI, 1.390–3.550; P = 0.001), WBC aOR 0.940 (95% CI, 0.907–0.973; P < 0.001), and CRP aOR 1.051 (95% CI, 0.911–1.214; P = 0.495) (S1 Appendix).

### Morphometric associations

In separately adjusted linear models, maximum diameter (aOR per 0.1 cm, 0.992; 95% CI, 0.861–1.143; P = 0.910), wall thickness (aOR per 0.1 cm, 0.875; 95% CI, 0.749–1.022; P = 0.091), and appendix length (aOR per 1 cm, 0.942; 95% CI, 0.876–1.013; P = 0.108) were not statistically significantly associated with complicated appendicitis (S1 Appendix). Exploratory analyses suggested possible nonlinearity for appendix length. In the combined complete-case morphometric model (n = 680), a centered quadratic appendix-length term remained significant (aOR, 1.049; 95% CI, 1.011–1.089; P = 0.012), whereas maximum diameter and wall thickness remained nonsignificant. Adding the morphometric block improved model fit only modestly (change in Nagelkerke R2, 0.019; likelihood-ratio P = 0.048). No case had Cook’s influence statistic greater than 1 or an absolute standardized residual greater than 3. The quadratic estimate remained similar after excluding 66 high-leverage observations (aOR, 1.046; 95% CI, 1.002–1.092; P = 0.040). Because these analyses were exploratory, used a reduced complete-case sample, and added limited explanatory information, they were not interpreted as establishing a clinically useful nonlinear marker.

## Discussion

This source-audited retrospective cohort of 1,010 histopathologically classified appendicitis encounters produced four principal findings. First, appendicolith was associated with approximately twofold higher adjusted odds of complicated appendicitis. Second, WBC demonstrated an unexpected inverse adjusted association rather than a positive association with greater disease severity. Third, the core model had weak discrimination and low explained variation, so it should be interpreted as an association model rather than a prediction tool. Fourth, the three recorded gross-pathology morphometric variables did not show statistically significant adjusted linear associations; an exploratory quadratic appendix-length signal was observed but added little explanatory information and requires external confirmation.

The contribution of this study is not the identification of appendicolith as a novel marker, but the provision of a source-audited estimate from a large pathology-confirmed Palestinian cohort and the joint evaluation of routinely documented clinical, laboratory, operative, postoperative, and gross-pathology variables.

The appendicolith finding is directionally consistent with histopathological and imaging-based studies linking appendicolith to more severe inflammation, gangrene, perforation, or treatment failure [13–15]. The effect estimate in the present cohort remained significant after adjustment for age, sex, WBC, and CRP. However, appendicolith was documented from routine operative and/or pathology records rather than from a standardized imaging protocol, and symptom duration was unavailable. The result therefore supports an association with severity but does not establish a causal pathway or prove that appendicolith should independently determine management.

The WBC result requires particular caution. Although WBC differed across the four severity categories, the pattern was not monotonic: the only significant adjusted pairwise difference was a lower distribution in gangrenous than suppurative appendicitis. The multivariable coefficient similarly indicated lower, not higher, odds of the binary complicated outcome with increasing WBC. Previous reviews and cohorts have shown that inflammatory biomarkers overlap substantially and that their independent performance varies with case mix, timing of sampling, symptom duration, and model specification [3,12]. In the present data, the inverse association should not be interpreted as protective or used to guide care. It may reflect unmeasured timing, prior treatment, selection among patients who underwent surgery, or residual confounding.

The modest model performance reinforces this restraint. An AUC of 0.594 and optimism-corrected AUC of 0.582 indicate weak individual-level discrimination, despite acceptable Hosmer-Lemeshow results. Models that incorporate detailed symptoms and imaging features have generally achieved better differentiation than models based only on a small set of routinely available demographic and laboratory variables [5–11]. The current model was intentionally parsimonious and chosen a priori, but it lacked symptom duration, temperature, examination findings, imaging features, and relevant comorbidities. Its value lies in estimating adjusted associations within this cohort, not in providing a clinical decision rule.

The gross-pathology morphometric findings should be interpreted cautiously. Maximum diameter, wall thickness, and appendix length did not show statistically significant linear adjusted associations, but the confidence intervals do not exclude small effects. The wall-thickness analysis was especially limited by 31.2% missingness. In addition, routine gross measurements may be influenced by fixation, tissue edema, handling, fragmentation, and measurement technique, none of which was consistently documented. Imaging-derived diameter and wall features, which capture the appendix in vivo and can incorporate surrounding inflammatory findings, are not directly comparable with gross-specimen measurements [5–7].

The exploratory nonlinear appendix-length result is hypothesis-generating. A quadratic term improved fit in the reduced complete-case cohort and remained similar after exclusion of high-leverage observations. Nevertheless, this analysis was prompted by diagnostic testing rather than prespecified as a primary hypothesis; the confidence interval approached the null in sensitivity analysis, and the incremental explained variation was small. Previous work has not shown a consistent simple relation between appendix length and perforation [16]. Accordingly, the present finding should be tested prospectively with standardized measurement and flexible modeling before any biological or clinical interpretation is made.

Operative approach, operative duration, and hospital stay differed across severity categories, but these variables should not be treated as baseline predictors. Operative approach may reflect surgeon preference, temporal practice patterns, patient characteristics, and anticipated disease severity. Longer operative duration in perforated cases is clinically plausible as a marker of greater technical complexity, whereas the significant omnibus hospital-stay result did not translate into any significant pairwise comparison after multiplicity adjustment. Postoperative wound infection and abscess were uncommon and showed no significant severity-category differences after exact testing. These outcome analyses were descriptive and were not sufficiently powered for robust multivariable modeling of rare events [18].

The study has several strengths. It includes a relatively large pathology-confirmed cohort from an underrepresented setting, uses an explicit four-category histopathological classification, reports variable-specific denominators, reconstructs one row per unique encounter from ID-linked source records, and provides effect estimates, confidence intervals, model diagnostics, internal validation, and prespecified sensitivity analyses. The comprehensive source audit strengthened the internal consistency, traceability, and reproducibility of the reported data.

The limitations are substantial. The retrospective, single-center design is susceptible to selection bias, information bias, and residual confounding. The cohort included only patients who underwent appendectomy and therefore cannot represent all patients with suspected or non-operatively managed appendicitis. Symptom duration, detailed physical findings, imaging features, preoperative antibiotic exposure, ASA class, diabetes, immunosuppression, body mass index, and other comorbidity measures were not consistently available. Laboratory timing relative to presentation and surgery could not be standardized. Appendicolith ascertainment depended on routine operative and/or pathology documentation rather than a uniform imaging protocol. Gross-pathology measurements were not collected under a documented research protocol, and fixation status, instrumentation, fragmentation handling, blinding, and interobserver reliability were not consistently available. Missingness was substantial for wall thickness and moderate for operative duration and the other morphometrics; complete-case sensitivity analyses may therefore be affected by selection. Although internal validation was performed, discrimination was weak and no external validation was undertaken. Finally, the nonlinear appendix-length analyses were exploratory and should not be considered confirmatory.

Future work should use prospective multicenter designs with standardized definitions of complicated appendicitis, detailed symptom chronology, preoperative imaging, treatment exposure, comorbidity data, and a prespecified specimen-measurement protocol. Studies should evaluate whether imaging-detected appendicolith and morphometric features add incremental discrimination and clinical utility beyond established clinical and laboratory variables, and any resulting prediction model should undergo appropriate internal and external validation.

## Conclusions

In this pathology-confirmed Palestinian cohort, appendicolith was associated with higher adjusted odds of complicated appendicitis, whereas WBC showed an unexpected inverse association that should not be interpreted as protective. The core model had weak discrimination and limited explained variation and is not suitable as a clinical prediction tool. Maximum diameter, wall thickness, and appendix length showed no statistically significant adjusted linear associations; an exploratory nonlinear appendix-length signal requires independent confirmation. These findings provide transparent regional estimates and underscore the need for prospective, standardized, multicenter evaluation that integrates symptom duration, comorbidities, imaging, laboratory data, and specimen measurements.

## Supporting information

S1 Appendix Supplementary statistical methods and tables. Includes variable missingness, adjusted pairwise comparisons, full model diagnostics and sensitivity analyses, morphometric effect estimates, and exploratory nonlinear morphometric analyses.
(DOCX)
