# Supplementary material for: Clinical, laboratory, operative, and appendiceal morphometric correlates of histopathological appendicitis severity: A retrospective cohort study from Palestine
Source: PLoS One. 2026 Sep 9;21(9):e0357811. doi: 10.1371/journal.pone.0357811 (PMC13557390; doi:10.1371/journal.pone.0357811)
Supplement: Supplementary file 1 [file pone.0357811.s001.docx]

S1 Appendix. Supplementary statistical methods and tables

**Clinical, laboratory, operative, and appendiceal morphometric correlates of histopathological appendicitis severity: a retrospective cohort study from Palestine**

**Ali Shakhshir^1,2†^, Mahdi Aljamal^3†^, Abdallah Alem^1^, Ibrahim Aljamal^4^, Zainah Amjad Issa^5^**

Table S1. Availability and missingness of study variables in the 1,010-patient cohort

| **Variable** | **Available, n** | **Missing, n** | **Missing, %** |
| --- | --- | --- | --- |
| Age, years | 1,010 | 0 | 0.0 |
| Sex | 1,009 | 1 | 0.1 |
| Operative approach | 1,010 | 0 | 0.0 |
| Nausea and/or vomiting | 940 | 70 | 6.9 |
| Other intraoperative finding | 1,006 | 4 | 0.4 |
| Appendicolith | 1,004 | 6 | 0.6 |
| CRP, mg/L | 998 | 12 | 1.2 |
| WBC, ×10⁹/L | 1,002 | 8 | 0.8 |
| Neutrophils, % | 1,000 | 10 | 1.0 |
| Operative duration, min | 900 | 110 | 10.9 |
| Hospital stay, days | 1,009 | 1 | 0.1 |
| Maximum diameter, cm | 903 | 107 | 10.6 |
| Wall thickness, cm | 695 | 315 | 31.2 |
| Appendix length, cm | 904 | 106 | 10.5 |
| Wound infection | 1,010 | 0 | 0.0 |
| Postoperative abscess | 1,010 | 0 | 0.0 |

No statistical imputation was performed. Descriptive and group-comparison analyses used available observations; regression analyses used complete cases for included variables.

Table S2. Bonferroni-adjusted pairwise comparisons after significant Kruskal-Wallis tests

| **Variable** | **Significant comparison** | **Adjusted P value** | **Direction/interpretation** |
| --- | --- | --- | --- |
| WBC, ×10⁹/L | Gangrenous vs suppurative | 0.002 | Lower in gangrenous |
| Neutrophils, % | Gangrenous vs perforated | 0.040 | Higher in perforated |
| Operative duration, min | Simple vs perforated | <0.001 | Longer in perforated |
| Operative duration, min | Gangrenous vs perforated | 0.006 | Longer in perforated |
| Operative duration, min | Suppurative vs perforated | 0.008 | Longer in perforated |
| Hospital stay, days | No pair remained significant | All >0.05 | Simple vs perforated adjusted P=0.052 |

Only significant adjusted comparisons are listed, except for hospital stay, for which no pair remained significant after Bonferroni adjustment.

Table S3A. Full coefficients from the primary adjusted association model

| **Variable** | **B** | **SE** | **Adjusted OR** | **95% CI** | **P value** |
| --- | --- | --- | --- | --- | --- |
| Appendicolith present (yes vs no) | 0.788 | 0.240 | 2.198 | 1.374-3.516 | 0.001 |
| WBC, per 1×10⁹/L | -0.064 | 0.018 | 0.938 | 0.906-0.971 | <0.001 |
| CRP, per 1 mg/L | 0.006 | 0.004 | 1.006 | 0.998-1.013 | 0.160 |
| Age, per year | 0.001 | 0.004 | 1.001 | 0.992-1.009 | 0.899 |
| Male sex (vs female) | -0.188 | 0.138 | 0.828 | 0.632-1.086 | 0.173 |

Analytic n=991; 320 complicated events. All covariates were selected a priori and entered simultaneously.

Table S3B. Primary model performance, assumptions, and internal validation

| **Metric** | **Estimate** | **Additional information** |
| --- | --- | --- |
| Analytic N (events) | 991 (320) | Complete cases |
| Likelihood-ratio chi-square | 26.895; df=5 | P<0.001 |
| Cox-Snell R2 | 0.027 |  |
| Nagelkerke R2 | 0.037 |  |
| Hosmer-Lemeshow chi-square | 4.717; df=8 | P=0.787 |
| AUC | 0.594 | 95% CI 0.557-0.632 |
| Bootstrap optimism-corrected AUC | 0.582 | 300 resamples |
| Brier score | 0.213 | Bootstrap 95% CI 0.202-0.224 |
| Bootstrap mean calibration intercept | -0.078 | 300 resamples |
| Bootstrap mean calibration slope | 0.891 | 300 resamples |
| VIF range | 1.004-1.035 | No meaningful multicollinearity |

AUC, area under the receiver operating characteristic curve; VIF, variance inflation factor. Bootstrap estimates used 300 resamples.

Table S3C. Sensitivity analysis with CRP modeled per doubling

| **Variable** | **Adjusted OR** | **95% CI** | **P value** |
| --- | --- | --- | --- |
| Age, per year | 1.001 | 0.992-1.009 | 0.873 |
| Male sex (vs female) | 0.833 | 0.635-1.092 | 0.185 |
| Appendicolith present | 2.221 | 1.390-3.550 | 0.001 |
| WBC, per 1×10⁹/L | 0.940 | 0.907-0.973 | <0.001 |
| CRP, per doubling | 1.051 | 0.911-1.214 | 0.495 |

Analytic n=991. Model likelihood-ratio chi-square=25.458 (df=5), P<0.001; Nagelkerke R2=0.035; Hosmer-Lemeshow P=0.533.

Table S4. Unadjusted and separately adjusted linear associations of gross-pathology morphometrics with complicated appendicitis

| **Variable** | **Unit** | **Unadjusted n** | **Unadjusted OR (95% CI)** | **P** | **Adjusted n** | **Adjusted OR (95% CI)** | **P** |
| --- | --- | --- | --- | --- | --- | --- | --- |
| Maximum diameter | Per 0.1 cm | 903 | 0.990 (0.862-1.138) | 0.891 | 890 | 0.992 (0.861-1.143) | 0.910 |
| Wall thickness | Per 0.1 cm | 695 | 0.881 (0.759-1.023) | 0.098 | 685 | 0.875 (0.749-1.022) | 0.091 |
| Appendix length | Per 1 cm | 904 | 0.951 (0.887-1.021) | 0.165 | 891 | 0.942 (0.876-1.013) | 0.108 |

Each adjusted model included age, male sex, appendicolith, WBC, and CRP. Separate models were used because variable-specific missingness produced different analytic samples.

Table S5A. Combined exploratory morphometric model performance

| **Metric** | **Estimate** | **P value/additional information** |
| --- | --- | --- |
| Complete-case N (events) | 680 (216) |  |
| Overall model chi-square | 25.610; df=9 | P=0.002 |
| Nagelkerke R2 | 0.052 |  |
| Hosmer-Lemeshow test | 5.095; df=8 | P=0.747 |
| Change vs core-only model | Δχ² = 9.592; df = 4; P = 0.048 | P=0.048 |
| Change in Nagelkerke R2 | 0.019 | 0.033 to 0.052 |

The combined model included the five core covariates, maximum diameter, wall thickness, centered appendix length, and the quadratic appendix-length term.

Table S5B. Morphometric terms in the combined exploratory model

| **Morphometric term** | **Adjusted OR** | **95% CI** | **P value** |
| --- | --- | --- | --- |
| Maximum diameter, per 0.1 cm | 0.990 | 0.823-1.190 | 0.914 |
| Wall thickness, per 0.1 cm | 0.888 | 0.754-1.044 | 0.151 |
| Appendix length centered at 6 cm | 0.961 | 0.888-1.041 | 0.333 |
| Quadratic appendix-length term | 1.049 | 1.011-1.089 | 0.012 |

Analytic n=680, including 216 complicated events. The quadratic term is exploratory and should not be interpreted as a validated clinical marker.

Table S5C. Influence diagnostics and high-leverage sensitivity analysis

| **Diagnostic/sensitivity result** | **Estimate** | **Interpretation** |
| --- | --- | --- |
| Appendix length range | 2.0-10.5 cm |  |
| Cook statistic >1 | 0/680 | Maximum 0.627 |
| Leverage >0.0294 | 66/680 (9.7%) | Maximum 0.256 |
| Absolute standardized residual >3 | 0/680 | Range -1.35 to 1.95 |
| High-leverage sensitivity cohort | 614 (190 events) |  |
| Quadratic term after excluding high leverage | 1.046 (1.002-1.092) | P=0.040 |

The high-leverage sensitivity analysis was used only to examine robustness of the quadratic appendix-length term. High-leverage observations were not excluded from the primary combined model.
