# Supplementary material for: Clinical, laboratory, operative, and appendiceal morphometric correlates of histopathological appendicitis severity: A retrospective cohort study from Palestine
Source: PLoS One. 2026 Sep 9;21(9):e0357811. doi: 10.1371/journal.pone.0357811 (PMC13557390; doi:10.1371/journal.pone.0357811)
Supplement: Supplementary file 2 [file pone.0357811.s003.docx]

Response to the Academic Editor and Reviewers

**Manuscript ID: PONE-D-26-13303**

*Clinical, laboratory, operative, and appendiceal morphometric correlates of histopathological appendicitis severity: a retrospective cohort study from Palestine*

**PLOS ONE**

________________________________________________________________________________

Dear Dr. Akhtar and Reviewers,

Thank you for the careful and constructive evaluation of our manuscript. We have undertaken a comprehensive revision that includes source-level cohort reconciliation, complete reanalysis, substantial rewriting of the Methods, Results, Discussion, and Conclusions, reconstruction and simplification of all main tables, addition of a participant-flow figure, and preparation of a detailed S1 Appendix. We believe these changes have substantially improved the accuracy, transparency, reproducibility, and clinical restraint of the manuscript.

Important note regarding the revised cohort and analyses. In direct response to the concerns regarding inconsistent denominators, we reconciled the original ID-linked clinical and morphometric source workbooks using Patient ID and date of surgery. This audit identified an analytic-file assembly problem in the legacy SPSS file, including duplicated encounter rows and eligible source records that had not been carried into that file. We therefore reconstructed the dataset so that it contained one row per unique patient encounter. Of 1,287 appendectomy encounters, 245 histologically normal appendices and 32 records without a classifiable severity category were excluded, yielding 1,010 unique eligible encounters. All descriptive statistics, hypothesis tests, regression models, model diagnostics, tables, the abstract, and manuscript interpretations were regenerated from the reconstructed dataset. The appendicolith association remained significant, while several numerical estimates and interpretations—most importantly the direction of the WBC association—were corrected.

Manuscript-location convention. Page and line references below refer to the latest revised manuscript. Supplementary material is cited by table number in the S1 Appendix.

# Summary of major revisions

- Reconstructed a unique-encounter cohort of 1,010 patients from the original ID-linked sources and added a participant-selection flow diagram.
- Reframed the manuscript around adjusted associations and regional/pathology-confirmed evidence rather than novelty of appendicolith or clinical prediction.
- Corrected the WBC directionality and removed any implication that higher WBC predicts greater severity or is protective.
- Rebuilt the statistical methods and analyses, including exact testing for sparse tables, adjusted pairwise comparisons, a priori covariate selection, functional-form assessment, VIFs, AUC, calibration, bootstrap internal validation, and sensitivity analyses.
- Reported variable-specific denominators and missingness, clarified complete-case analysis, and added Table S1.
- Added effect estimates and 95% confidence intervals for morphometric variables and carefully qualified exploratory nonlinear appendix-length findings.
- Rebuilt and simplified all main tables, added S1 Appendix tables, converted the abstract to an unstructured format, corrected citation order and duplicate references, and clarified ethics and controlled data access.

# Journal requirements

**Comment:** Please ensure that your manuscript meets PLOS ONE style requirements, including those for file naming.

**Response:** We revised the manuscript structure and presentation to conform closely to PLOS ONE formatting. We prepared a clean manuscript, a marked manuscript with all revised/new text shown in red, a separately uploaded main figure (Fig 1), and a clean and marked S1 Appendix. The abstract was converted to a single unstructured paragraph, remains below the 300-word limit, and defines nonstandard abbreviations at first use. Figure citations and captions are retained in the manuscript while the figure is supplied as a separate high-resolution file. Tables were simplified, white blood cell count units and statistical notation were standardized, and the reference list was reformatted, de-duplicated, and renumbered in order of first citation.

**Location in revised files:** Throughout the revised manuscript and submission package.

**Comment:** The data-access contact must not be an author and must be an institutional contact. Please revise the data statement and include contact information for the third-party organization.

**Response:** We replaced author-dependent access with an institutional controlled-access pathway. The revised statement identifies the Arab American University Institutional Review Board and Al-Makassed Islamic Charitable Society Hospital as the non-author institutional contacts, provides contact details for both institutions, explains that requests will be reviewed under applicable ethical and legal requirements and a data-use agreement, and states that the authors had no special access privileges. The underlying patient-level data are held in institutional medical and pathology records rather than a public repository; therefore, a repository dataset citation is not applicable.

**Location in revised files:** Main manuscript, Data availability, page 16, lines 355-363.

**Comment:** The ethics statement should appear only in the Methods section.

**Response:** The ethics statement is now located only in the Materials and methods section. It states the exact IRB reference and approval date (8 July 2025), explains that the Arab American University IRB provided ethical oversight and that Al-Makassed Hospital provided institutional permission for record access, documents the consent waiver, and describes de-identified data handling. Any duplicate ethics wording outside the Methods was removed.

**Location in revised files:** Main manuscript, Ethics statement, pages 7-8, lines 165-174.

**Comment:** If reviewer comments recommend specific publications, evaluate their relevance; citation is not mandatory unless directed by the editor.

**Response:** No reviewer-mandated citation was added automatically. We independently reviewed and updated the literature to support the revised knowledge-gap framing, statistical interpretation, appendicolith discussion, morphometric limitations, and reporting approach. Duplicate references were removed and bibliographic details and DOIs were checked.

**Location in revised files:** Main manuscript, Introduction and Discussion; revised References, pages 17-18, lines 377-425.

# Academic Editor

**Comment:** “It is a study on a common subject... [the] study does not add anything new to the existing evidence nor identify any gaps in knowledge... [and] has a number of inconsistencies in numbers as well as interpretation of statistical tests.”

**Response:** We agree that the original manuscript did not adequately distinguish its contribution from the extensive existing appendicitis literature and that the numerical and interpretive inconsistencies substantially weakened the work. We therefore reframed the manuscript and performed a full source-level and statistical reconstruction rather than attempting isolated textual corrections.

The revised Introduction now explicitly states that the contribution is not the discovery of appendicolith as a new risk factor. Instead, the manuscript is positioned as a large, pathology-confirmed, source-audited Palestinian cohort that integrates routinely recorded clinical, laboratory, operative, postoperative, and gross-pathology variables; quantifies morphometric associations with confidence intervals; and reports model performance and internal validation. The title and manuscript terminology consistently use “correlates” and “associations,” and the regression is explicitly described as an association model rather than a prediction rule.

To resolve the numerical deficiencies, we reconciled the ID-linked source workbooks, reconstructed one row per unique encounter, established a transparent participant flow, regenerated every analysis, rebuilt all tables, and added variable-specific denominators. The WBC interpretation was corrected to an unexpected inverse association, CRP was no longer described as significantly different across severity categories, exact tests and adjusted post-hoc comparisons were used where appropriate, and all conclusions were rewritten to match the corrected results.

The revised Discussion and Conclusion now emphasize regional estimates, transparent negative findings, weak model discrimination, residual confounding, measurement limitations, and the need for prospective multicenter confirmation rather than overclaiming novelty or clinical utility.

**Location in revised files:** Main manuscript: Introduction, page 3, lines 58-71; cohort reconstruction, pages 4 and 8, lines 79-95 and 176-182; statistical methods, pages 6-7, lines 131-164; corrected Results, pages 9-12, lines 197-258; Discussion and Conclusions, pages 12-16, lines 259-354. Fig 1 and S1 Appendix added.

# Reviewer #1

**Comment:** “This study highlights... observations and evidence already available in literature. But it could be of significance in the local/national context.”

**Response:** Thank you for recognizing the value of the cohort and its potential local and national relevance. We agree that the manuscript should not present established appendicitis associations as novel. The revised Introduction now explicitly acknowledges the established literature and states that the study’s contribution lies in providing a source-audited, pathology-confirmed estimate from a large Palestinian hospital cohort, together with transparent evaluation of routinely recorded gross-pathology morphometrics and comprehensive reporting of model limitations. The Discussion similarly positions the findings as contextual regional evidence and avoids claims that the appendicolith association is new.

**Location in revised files:** Main manuscript, Introduction, page 3, lines 58-71; Discussion, pages 12-15, lines 259-344; Conclusions, page 16, lines 345-354.

# Reviewer #2

**Comment:** Overall assessment: the study has value because of its large cohort and pathology-confirmed outcomes, but methodological, analytical, and reporting issues require revision.

**Response:** We appreciate this balanced assessment. We have undertaken a comprehensive revision that addresses each methodological, analytical, and reporting concern. The cohort was reconstructed from ID-linked source data, all analyses were repeated, the manuscript was reframed as an association study, all main tables were rebuilt, a detailed S1 Appendix was added, and interpretation was made substantially more cautious.

**Location in revised files:** Throughout the revised manuscript and S1 Appendix.

**Comment:** Consistent data reporting: “N discrepancies... Provide a single clear CONSORT-style flowchart: total appendectomies identified, exclusions (with reasons and numbers), final N and per-variable denominators.”

**Response:** We performed a source-level reconciliation specifically to address the denominator inconsistencies. The original clinical source contained 1,287 unique Patient ID-surgery date encounters. We excluded 245 histologically normal appendices and 32 records without a classifiable histopathological severity category, yielding a final cohort of 1,010 unique encounters. The source audit identified duplicated encounter rows in the legacy analytic file and eligible source records that had not been carried into that file; the reconstructed dataset therefore uses one row per unique encounter. All analyses and tables were rerun using this cohort.

We added Fig 1, a participant-selection and cohort-reconstruction diagram, and report exact available N values in the text and tables. Table 1 and Table 2 show variable-specific denominators; Table S1 reports available and missing counts and percentages for every major variable. Percentages are explicitly calculated using available observations.

**Location in revised files:** Main manuscript, Participants and cohort reconstruction, page 4, lines 79-95; Results and Fig 1, page 8, lines 176-190; Tables 1-2, pages 8-9; S1 Appendix, Table S1.

**Comment:** Multivariable model: “Adjusted OR for WBC = 0.93... implies higher WBC is protective, yet the text states elevated WBC independently predicts complicated appendicitis. Reconcile directionality, coding and units, and correct text.”

**Response:** We agree. The original interpretation was incorrect. We verified the outcome coding (0=uncomplicated; 1=complicated), confirmed the WBC units, and reran the model using the reconstructed dataset. The corrected adjusted association is inverse: aOR 0.938 per 1 × 10⁹/L increase (95% CI 0.906-0.971; P<0.001). The abstract, Results, Discussion, tables, and Conclusion have all been corrected.

We do not describe WBC as protective. The revised manuscript calls the association unexpected and emphasizes that it may reflect unmeasured timing, prior treatment, surgical selection, case mix, or residual confounding. The four-category analyses also showed a non-monotonic pattern, with the significant adjusted pairwise difference occurring between gangrenous and suppurative disease.

**Location in revised files:** Main manuscript, Abstract, page 2, lines 18-41; primary model, page 11, lines 223-243 and Table 5; Discussion, pages 13-14, lines 276-284; Conclusions, page 16, lines 345-354; S1 Appendix, Tables S2, S3A, and S3C.

**Comment:** Model performance: “Present discrimination (AUC/ROC), calibration... and internal validation... if claiming predictive value.”

**Response:** We substantially expanded model-performance reporting and, importantly, removed predictive claims. The regression is now explicitly described as an adjusted association model rather than a clinical prediction tool. We report the likelihood-ratio chi-square, Cox-Snell and Nagelkerke R², Hosmer-Lemeshow test, AUC with 95% CI, Brier score, bootstrap optimism-corrected AUC, and bootstrap mean calibration intercept and slope. Internal validation used 300 nonparametric bootstrap resamples.

The corrected model had weak discrimination (AUC 0.594, 95% CI 0.557-0.632; optimism-corrected AUC 0.582) and low explained variation (Nagelkerke R² 0.037). These limitations are prominent in the Abstract, Results, Discussion, and Conclusion, and we explicitly state that the model should not be used as a clinical prediction tool.

**Location in revised files:** Main manuscript, Statistical analysis, pages 6-7, lines 141-164; Table 5 and model-performance Results, pages 11-12, lines 223-243; Discussion, pages 13-14, lines 285-292; Conclusions, page 16, lines 345-354; S1 Appendix, Table S3B.

**Comment:** Predictor selection, multicollinearity, functional form, and cutoffs: explain candidate-predictor selection, report VIFs, clarify how continuous variables were modeled, and consider transformations/cutoffs and sensitivity analyses.

**Response:** The revised Methods now state that age, sex, appendicolith, WBC, and CRP were selected a priori on the basis of clinical relevance, availability, and prior literature and were entered simultaneously; automated stepwise selection was not used. Operative approach, duration, and hospital stay were excluded from the core model because they may represent treatment selection or consequences of severity rather than baseline factors.

Continuous variables were retained in their original units to preserve information and avoid arbitrary data-derived cutoffs. Linearity in the logit was assessed using Box-Tidwell interaction terms with a Bonferroni-adjusted threshold. No core predictor crossed the adjusted threshold. Multicollinearity was assessed using tolerance and VIF; VIFs ranged from 1.004 to 1.035, indicating no meaningful multicollinearity. Because CRP showed a borderline unadjusted functional-form signal and is right-skewed, we added a sensitivity analysis with CRP on the log₂ scale, interpreted per doubling; the substantive findings were unchanged.

**Location in revised files:** Main manuscript, Statistical analysis, pages 6-7, lines 141-164; model diagnostics and sensitivity analyses, pages 11-12, lines 234-243; S1 Appendix, Tables S3B and S3C.

**Comment:** Missing important covariates and confounders: symptom duration, imaging, preoperative antibiotics, ASA class, immunosuppression, diabetes, and comorbidities should be included if available or acknowledged as limitations.

**Response:** These variables were not consistently available in the retrospective source records and could not be added reliably. We now state this explicitly in the Methods and explain that detailed imaging findings, symptom duration, preoperative antibiotic exposure, ASA class, diabetes, immunosuppression, body mass index, and other comorbidity measures were not included because of inconsistent availability. We also added these omissions prominently to the limitations and explain how they may contribute to residual confounding and variable biomarker performance.

We further note that appendicolith ascertainment was based on routine operative and/or pathology documentation rather than a uniform imaging protocol, and that laboratory timing relative to presentation and surgery could not be standardized.

**Location in revised files:** Main manuscript, Data sources and study variables, page 5, lines 96-105; Discussion limitations, pages 14-15, lines 324-338; future-work recommendations, pages 15-16, lines 339-344.

**Comment:** Measurement and interpretation of morphometrics: discuss fixation, specimen handling, fragmentation, reliability, measurement personnel/blinding, imaging comparison, units, and present effect estimates with confidence intervals rather than simply stating limited predictive value.

**Response:** We revised the morphometric Methods to report only what is supported by the available records. Maximum diameter, wall thickness, and appendix length were abstracted in centimeters from routine gross-pathology information. We clarify that these were routine clinical record measurements, not prospectively standardized research measurements. The available records did not consistently document fixation timing, measuring instrument, handling of fragmented specimens, blinding, or interobserver reliability; therefore, we removed any unsupported implication that these procedures were standardized. Imaging-derived morphometrics were not systematically available and could not be compared with gross-specimen measurements.

We confirmed that the recorded unit was centimeters; the median maximum diameter of 0.5 cm is therefore equivalent to 5 mm. The manuscript now treats the coarse measurement distribution and uncertain protocol as limitations rather than overinterpreting them.

We added unadjusted and separately adjusted odds ratios with 95% CIs and analytic N values for all three morphometric variables. Maximum diameter, wall thickness, and appendix length had no statistically significant adjusted linear associations. Because diagnostic assessment suggested possible curvature for appendix length, we also report carefully labeled exploratory quadratic sensitivity analyses. These analyses added little explanatory information and are presented as hypothesis-generating only, not as evidence of a validated predictor.

**Location in revised files:** Main manuscript, Gross-pathology morphometric variables, pages 5-6, lines 113-121; Statistical analysis, page 7, lines 154-160; Morphometric Results, page 12, lines 244-258; Discussion, pages 14-15, lines 293-308; S1 Appendix, Tables S4 and S5A-S5C.

**Comment:** Tables and results: “Numerous formatting and content issues... Include N for each variable, present medians (IQR) and p-values where appropriate, and ensure all percentages sum correctly.”

**Response:** All main tables were rebuilt from the reconstructed dataset and simplified while retaining a layout close to the original manuscript. Table 1 reports categorical characteristics with explicit denominators; Table 2 reports overall medians and IQRs with N values; Table 3 reports four-category counts and column percentages with corrected exact/chi-square P values; Table 4 reports medians (IQR) and Kruskal-Wallis P values; and Table 5 reports the primary adjusted model with coefficients, standard errors, aORs, 95% CIs, and P values. Footnotes now specify denominators, test choice, missing-data handling, and abbreviations.

We verified all totals and percentages against the reconstructed source. Detailed missingness, pairwise comparisons, model diagnostics, sensitivity analyses, and morphometric estimates were moved to the S1 Appendix to keep the main tables concise.

**Location in revised files:** Main manuscript, Tables 1-5, pages 8-11; S1 Appendix, Tables S1-S5.

**Comment:** References: verify bibliographic information and remove duplicate references [5]/[11] and [9]/[23].

**Response:** We reviewed and rebuilt the reference list. The duplicate Lin et al. citation and duplicate Li et al. citation were removed. Bibliographic details, journal names, years, volumes, article/page numbers, and DOIs were checked and standardized. We also corrected the final first-citation sequence by placing the STROBE statement as reference [17] and the Son et al. postoperative-abscess study as reference [18]. The revised reference list contains 18 unique references numbered in the order in which they first appear in the text.

**Location in revised files:** Main manuscript, References, pages 17-18, lines 377-425.

**Comment:** Missing data: report how missing data were handled and the proportion missing for each variable.

**Response:** We added a dedicated Data quality and missing-data handling subsection. Raw source values were preserved in the audit file. Five WBC values below 1.0 × 10⁹/L and six neutrophil values below 1% were treated as missing in the cleaned analysis variables because they were incompatible with the stated units and could not be verified. One isolated wall-thickness value of 3.0 cm was treated as missing rather than changed to an assumed value. No imputation was performed. Descriptive and group-comparison analyses used available observations, whereas each regression used complete cases for the variables included.

Table S1 now reports available N, missing N, and missing percentage for each major variable. The main text also highlights substantial wall-thickness missingness (31.2%) and discusses possible complete-case selection in the limitations.

**Location in revised files:** Main manuscript, Data quality and missing-data handling, page 6, lines 122-130; Results, pages 8-9, lines 184-195; Discussion limitations, pages 14-15, lines 324-338; S1 Appendix, Table S1.

**Comment:** Ethics: explain the Arab American University–Al-Makassed jurisdictional arrangement and provide the approval date and reference.

**Response:** We revised the Ethics statement to provide the exact IRB reference (J-2025/A/2026/N) and approval date (8 July 2025). We explain that the Arab American University Institutional Review Board provided ethical oversight for the research and that institutional permission to access the records was obtained from Al-Makassed Islamic Charitable Society Hospital, the data-holding clinical site. The statement also records the waiver of individual informed consent and de-identified data handling.

**Location in revised files:** Main manuscript, Ethics statement, pages 7-8, lines 165-174.

# Closing statement

We thank the Academic Editor and both reviewers again for their detailed and constructive comments. The review prompted a source-level audit and a substantially more rigorous reconstruction of the study. We believe the revised manuscript now presents a transparent participant flow, internally consistent denominators, appropriately selected and diagnosed association models, corrected statistical interpretation, complete missing-data reporting, quantified morphometric effects, a restrained discussion of limitations and exploratory findings, and a clearer statement of the study’s regional and methodological contribution. We hope that the revised manuscript is now suitable for further consideration by PLOS ONE.

Sincerely,

Ali Shakhshir, MD, and Mahdi Aljamal, MD
On behalf of all authors
