# Supplementary material for: Clinical, laboratory, operative, and appendiceal morphometric correlates of histopathological appendicitis severity: A retrospective cohort study from Palestine
Source: PLoS One. 2026 Sep 9;21(9):e0357811. doi: 10.1371/journal.pone.0357811 (PMC13557390; doi:10.1371/journal.pone.0357811)
Supplement: Supplementary file 3 [file pone.0357811.s004.docx]

Response to the Academic Editor

**Manuscript ID: PONE-D-26-13303R1**

Clinical, laboratory, operative, and appendiceal morphometric correlates of histopathological appendicitis severity: a retrospective cohort study from Palestine

PLOS ONE

________________________________________________________________________________

Dear Dr. Akhtar,

Thank you for the opportunity to revise our manuscript further. We have addressed each point raised in the current minor-revision decision. No new reviewer comments were included in the current decision letter. The revision is focused on presentation and transparency: the abstract has been converted to a structured format, the Introduction has been shortened and interpretive appendicolith material has been moved to the Discussion, and the handling and presentation of variable-specific denominators have been clarified in the Methods, Results, and Tables 1-4. No statistical analyses, cohort definitions, or numerical results were changed.

All changes made in response to this decision are shown in red in the marked manuscript. Page and line references below refer to that marked manuscript.

# Journal requirements

Comment 1: If reviewer comments recommend specific previously published works, please evaluate their relevance; citation is not mandatory unless directed by the editor.

Response: No specific additional publication was recommended in the current decision. We therefore did not add citations solely for compliance. The references retained in the manuscript were reviewed for relevance to the study rationale, methods, and interpretation.

Location in revised manuscript: References, pages 17-18, lines 382-430.

Comment 2: Please review the reference list to ensure that it is complete and correct and address any retracted publications.

Response: We rechecked the complete reference list, including author names, article titles, journals, years, volumes, pages or article numbers, and DOI information. None of the cited publications is identified as retracted, and no retracted article is cited. No reference-list change was required in this revision.

Location in revised manuscript: References, pages 17-18, lines 382-430.

# Additional Academic Editor comments

Comment 1: “Abstract may be converted into a structured format keeping word count upfront. Each section should provide objective information rather than vague statements.”

Response: We agree. The abstract has been converted to a structured format with the headings Background, Methods, Results, and Conclusions. The revised abstract contains 270 words excluding the section labels and remains below the journal limit. Each section now provides objective information: the Background states the specific evidence gap and objective; the Methods report the setting, cohort selection, outcome definition, comparison methods, and prespecified model; the Results report the category counts, significant group differences, adjusted effect estimates, confidence intervals, P values, model discrimination, and morphometric findings; and the Conclusions provide a restrained interpretation without predictive claims.

Location in revised manuscript: Abstract, page 2, lines 17-41.

Comment 2: “Introduction in present format contains information like that related to the appendicolith which is not appropriate. Line 64 and onwards are out of place. This may be moved to discussion section.”

Response: We agree. The detailed appendicolith interpretation and the prior defensive contribution statement have been removed from the Introduction. The final Introduction now contains a concise evidence-gap paragraph and the study objectives only. The study-contribution statement has been moved to the Discussion, where it is presented immediately before the detailed interpretation of the appendicolith finding.

Location in revised manuscript: Introduction, page 3, lines 58-64; Discussion, page 13, lines 270-280.

Comment 3: “In results section, readers may find it difficult to comprehend the information as for each variable denominator is different... This may be looked into. It also raises question as to how statistical analysis was done for each category of variables.”

Response: We agree and have revised both the analytical explanation and table presentation. The Methods now state explicitly that no values were imputed; each four-category comparison was performed separately using all nonmissing observations for the variable being analyzed; and patients were not excluded from other analyses solely because another variable was missing. Regression models used complete cases for the variables included in the respective model. The Results now alert readers to variable-specific availability before the first table. Tables 1 and 2 continue to display the available N beside each variable. Tables 3 and 4 now also display the available overall N beside every variable and the total size of each histopathological category in the column headings. Their footnotes explain precisely how percentages and continuous summaries were calculated and state that no imputation was performed. For clarity, operative approach was complete for all 1,010 encounters, whereas sex was available for 1,009 encounters and appendicolith status for 1,004 encounters. The full variable-level missingness table remains in S1 Appendix.

Location in revised manuscript: Data quality and missing-data handling, pages 5-6, lines 115-126; Overall cohort characteristics, page 8, lines 179-188; Tables 1-2, pages 8-9; Tables 3-4 and revised footnotes, pages 10-11, lines 203-222; S1 Appendix, Table S1.

We appreciate the Academic Editor’s focused guidance. We believe these changes improve the readability of the manuscript and make the analytical denominators and missing-data handling immediately clear without altering the underlying study results.

Sincerely,

**Ali Shakhshir, MD**

On behalf of all authors
