# Supplementary material for: Clinical, laboratory, operative, and appendiceal morphometric correlates of histopathological appendicitis severity: A retrospective cohort study from Palestine
Source: PLoS One. 2026 Sep 9;21(9):e0357811. doi: 10.1371/journal.pone.0357811 (PMC13557390; doi:10.1371/journal.pone.0357811)
Supplement: Peer review history

# Peer review: Decision Letter 0

**DOI:** 10.1371/journal.pone.0357811.r001

**Authors:** Jamshed Akhtar

22 Jun 2026

Dear Dr. Shakhshir,

Thank you for submitting your manuscript to PLOS ONE. After careful consideration, we feel that it has merit but does not fully meet PLOS ONE’s publication criteria as it currently stands. Therefore, we invite you to submit a revised version of the manuscript that addresses the points raised during the review process.

Please submit your revised manuscript by Aug 06 2026 11:59PM. If you will need more time than this to complete your revisions, please reply to this message or contact the journal office at plosone@plos.org. When you're ready to submit your revision, log on to https://www.editorialmanager.com/pone/ and select the 'Submissions Needing Revision' folder to locate your manuscript file.

- A letter that responds to each point raised by the academic editor and reviewer(s). You should upload this letter as a separate file labeled 'Response to Reviewers'.
- A marked-up copy of your manuscript that highlights changes made to the original version. You should upload this as a separate file labeled 'Revised Manuscript with Track Changes'.
- An unmarked version of your revised paper without tracked changes. You should upload this as a separate file labeled 'Manuscript'.

If you would like to make changes to your financial disclosure, please include your updated statement in your cover letter. Guidelines for resubmitting your figure files are available below the reviewer comments at the end of this letter.

If applicable, we recommend that you deposit your laboratory protocols in protocols.io to enhance the reproducibility of your results. Protocols.io assigns your protocol its own identifier (DOI) so that it can be cited independently in the future. For instructions see: https://journals.plos.org/plosone/s/submission-guidelines#loc-laboratory-protocols. Additionally, PLOS ONE offers an option for publishing peer-reviewed Lab Protocol articles, which describe protocols hosted on protocols.io. Read more information on sharing protocols at https://plos.org/protocols?utm_medium=editorial-email&utm_source=authorletters&utm_campaign=protocols.

As the corresponding author, your ORCID iD is verified in the submission system and will appear in the published article. PLOS supports the use of ORCID, and we encourage all coauthors to register for an ORCID iD and use it as well. Please encourage your coauthors to verify their ORCID iD within the submission system before final acceptance, as unverified ORCID iDs will not appear in the published article. *Only*  the individual author can complete the verification step; PLOS staff *cannot*  verify ORCID iDs on behalf of authors.

We look forward to receiving your revised manuscript.

Kind regards,

Jamshed Akhtar, MBBS, FCSP, FRCS, MHPE, FACS, M Bioethics

Academic Editor

PLOS One

Journal Requirements:

When submitting your revision, we need you to address these additional requirements.

1. Please ensure that your manuscript meets PLOS ONE's style requirements, including those for file naming. The PLOS ONE style templates can be found at

https://journals.plos.org/plosone/s/file?id=wjVg/PLOSOne_formatting_sample_main_body.pdf and

https://journals.plos.org/plosone/s/file?id=ba62/PLOSOne_formatting_sample_title_authors_affiliations.pdf.

2. In the online submission form you indicate that your data is not available for proprietary reasons and have provided a contact point for accessing this data. Please note that your current contact point is a co-author on this manuscript. According to our Data Policy, the contact point must not be an author on the manuscript and must be an institutional contact, ideally not an individual. Please revise your data statement to a non-author institutional point of contact, such as a data access or ethics committee, and send this to us via return email. Please also include contact information for the third party organization, and please include the full citation of where the data can be found.

3.Your ethics statement should only appear in the Methods section of your manuscript. If your ethics statement is written in any section besides the Methods, please delete it from any other section.

4. If the reviewer comments include a recommendation to cite specific previously published works, please review and evaluate these publications to determine whether they are relevant and should be cited. There is no requirement to cite these works unless the editor has indicated otherwise.

Additional Editor Comments (if provided):

It is a study on a common subject. Acute appendicitis is a common surgical emergency and various aspects related to it are well reported in literature. As such study does not add anything new to the existing evidence neither identify any gaps in Knowledge that were to be addressed. The current manuscript has number of inconsistencies in numbers as well as interpretation of statistical tests. All these deficiencies limit the usefulness of data. Processing such an article is not possible in present form.

[Note: HTML markup is below. Please do not edit.]

Reviewers' comments:

Reviewer's Responses to Questions

**Comments to the Author**

1. Is the manuscript technically sound, and do the data support the conclusions?

Reviewer #1: Yes

Reviewer #2: Partly

**********

2. Has the statistical analysis been performed appropriately and rigorously?

Reviewer #1: Yes

Reviewer #2: Yes

**********

3. Have the authors made all data underlying the findings in their manuscript fully available?

The PLOS Data policy

Reviewer #1: Yes

Reviewer #2: Yes

**********

4. Is the manuscript presented in an intelligible fashion and written in standard English?

Reviewer #1: Yes

Reviewer #2: Yes

**********

Reviewer #1: I appreciate the efforts placed in collecting data and writing manuacript based on that data of a very common and frequently encountered problem. This study highlights the same. Observations and eveidence already available in literature. But it could be of aignificane in the the local/ national context.

Reviewer #2: Manuscript title: predictors of complicated acute appendicitis — retrospective cohort, Al‑Makassed Hospital, Palestine

This is a relatively large single‑center retrospective series (n ≈ 997) assessing morphometric, clinical, laboratory and operative correlates of histopathologic appendicitis severity. The authors’ main claim is that the presence of appendicolith and higher WBC independently predict complicated appendicitis; morphometric measures have limited value.

Revision before further consideration is recommended. The study has value (large cohort, pathology‑confirmed outcomes) but several methodological, analytical, and reporting issues could be addressed. The following could be addressed to make this paper stronger:

Consistent data reporting:

N discrepancies: manuscript alternately reports denominators of 996, 997, 991, 988, etc. (e.g., Table 1 header “Gender (N: 996)” but text says 997). Provide a single clear CONSORT‑style flowchart: total appendectomies identified, exclusions (with reasons and numbers), final N and per‑variable denominators.

Statistical analysis and interpretation:

Multivariable model results need clarification. Reported adjusted OR for WBC = 0.93 (95% CI 0.90–0.97, p<0.001) implies higher WBC is protective, yet text states elevated WBC independently predicts complicated appendicitis. Reconcile directionality, coding and units, and correct text.

Model performance could be strengthened (Nagelkerke R2 = 0.052). Authors could present discrimination (AUC/ROC), calibration (calibration plot or Hosmer‑Lemeshow), and internal validation (bootstrap or cross‑validation) if claiming predictive value.

Authors could explain how candidate predictors were selected (clinical a priori vs p‑value threshold). Consider multicollinearity (CRP vs WBC) and reporting Variance Inflation Factors (VIFs) to measure the severity of multicollinearity (high correlation) between predictor variables in the multiple regression model. The authors could provide whether continuous variables were modeled linearly or transformed and justify choices; consider clinically meaningful cutoffs and sensitivity analyses.

Missing important covariates and potential confounders:

Symptom duration (time from onset to presentation) is a known strong predictor of complicated appendicitis but is not reported/adjusted for. If available, consider including this; if not available, acknowledge as a key limitation. Imaging findings (CT/US) and preoperative management (e.g. antibiotics prior to surgery) are not included but could confound appendicolith detection and outcomes— similarly, the authors could report availability or absence as a limitation.

Comorbidities (ASA class, immunosuppression, diabetes) are not addressed—these affect perforation risk and should be considered or discussed as limitations.

Measurement and interpretation of morphometrics:

Gross pathology measurements may be influenced by fixation, specimen handling and fragmentation. Authors could discuss measurement bias, reliability (who measured, interobserver checks), and why imaging morphometrics (when available) were not used or compared. Clarify units (appendiceal diameter median 0.5 cm = 5 mm seems small — confirm measurement units and method). State clearly who performed pathology measurements (pathologists blinded to clinical severity?), how fragmented specimens were handled statistically. The claim that morphometrics “showed limited predictive value” should be nuanced: present effect estimates for these variables and negative results with CIs so readers can judge clinically meaningful differences.

Tables and results:

Numerous formatting and content issues in tables (misplaced numbers, unclear denominators, inconsistent p‑value footnotes). Include N for each variable, present medians (IQR) and p-values where appropriate, and ensure all percentages sum correctly.

References:

Verify and update all references to correct journal, year, volume, pages, and DOIs. Avoid duplicates:

• References [5] and [11]: both cite H. A. Lin et al., World Journal of Emergency Surgery 2021 (same title and details).

• References [9] and [23]: both cite D. Li et al., Scientific Reports (Sci. Rep.) 2025 (same title and details).

Missing data:

Report how missing data were handled (complete case analysis vs imputation). Report proportion of missing values per variable.

Ethics:

If the study site is Al‑Makassed Hospital but IRB is Arab American University, explain the IRB jurisdictional arrangement and include IRB approval date and reference clearly.

**********

what does this mean?). If published, this will include your full peer review and any attached files.

If you choose “no”, your identity will remain anonymous but your review may still be made public.

**Do you want your identity to be public for this peer review?**  For information about this choice, including consent withdrawal, please see our Privacy Policy

Reviewer #1: No

Reviewer #2: No

**********

[NOTE: If reviewer comments were submitted as an attachment file, they will be attached to this email and accessible via the submission site. Please log into your account, locate the manuscript record, and check for the action link "View Attachments". If this link does not appear, there are no attachment files.]

To ensure your figures meet our technical requirements, please review our figure guidelines: https://journals.plos.org/plosone/s/figures

You may also use PLOS’s free figure tool, NAAS, to help you prepare publication quality figures: https://journals.plos.org/plosone/s/figures#loc-tools-for-figure-preparation.

NAAS will assess whether your figures meet our technical requirements by comparing each figure against our figure specifications.

# Author comment: Author response to Decision Letter 1

**DOI:** 10.1371/journal.pone.0357811.r002

14 Jul 2026

Response to the Academic Editor and Reviewers

Manuscript ID: PONE-D-26-13303

Clinical, laboratory, operative, and appendiceal morphometric correlates of histopathological appendicitis severity: a retrospective cohort study from Palestine

PLOS ONE

________________________________________________________________________________

Dear Dr. Akhtar and Reviewers,

Thank you for the careful and constructive evaluation of our manuscript. We have undertaken a comprehensive revision that includes source-level cohort reconciliation, complete reanalysis, substantial rewriting of the Methods, Results, Discussion, and Conclusions, reconstruction and simplification of all main tables, addition of a participant-flow figure, and preparation of a detailed S1 Appendix. We believe these changes have substantially improved the accuracy, transparency, reproducibility, and clinical restraint of the manuscript.

Important note regarding the revised cohort and analyses. In direct response to the concerns regarding inconsistent denominators, we reconciled the original ID-linked clinical and morphometric source workbooks using Patient ID and date of surgery. This audit identified an analytic-file assembly problem in the legacy SPSS file, including duplicated encounter rows and eligible source records that had not been carried into that file. We therefore reconstructed the dataset so that it contained one row per unique patient encounter. Of 1,287 appendectomy encounters, 245 histologically normal appendices and 32 records without a classifiable severity category were excluded, yielding 1,010 unique eligible encounters. All descriptive statistics, hypothesis tests, regression models, model diagnostics, tables, the abstract, and manuscript interpretations were regenerated from the reconstructed dataset. The appendicolith association remained significant, while several numerical estimates and interpretations—most importantly the direction of the WBC association—were corrected.

Manuscript-location convention. Page and line references below refer to the latest revised manuscript. Supplementary material is cited by table number in the S1 Appendix.

Summary of major revisions

• Reconstructed a unique-encounter cohort of 1,010 patients from the original ID-linked sources and added a participant-selection flow diagram.

• Reframed the manuscript around adjusted associations and regional/pathology-confirmed evidence rather than novelty of appendicolith or clinical prediction.

• Corrected the WBC directionality and removed any implication that higher WBC predicts greater severity or is protective.

• Rebuilt the statistical methods and analyses, including exact testing for sparse tables, adjusted pairwise comparisons, a priori covariate selection, functional-form assessment, VIFs, AUC, calibration, bootstrap internal validation, and sensitivity analyses.

• Reported variable-specific denominators and missingness, clarified complete-case analysis, and added Table S1.

• Added effect estimates and 95% confidence intervals for morphometric variables and carefully qualified exploratory nonlinear appendix-length findings.

• Rebuilt and simplified all main tables, added S1 Appendix tables, converted the abstract to an unstructured format, corrected citation order and duplicate references, and clarified ethics and controlled data access.

Journal requirements

Comment: Please ensure that your manuscript meets PLOS ONE style requirements, including those for file naming.

Response: We revised the manuscript structure and presentation to conform closely to PLOS ONE formatting. We prepared a clean manuscript, a marked manuscript with all revised/new text shown in red, a separately uploaded main figure (Fig 1), and a clean and marked S1 Appendix. The abstract was converted to a single unstructured paragraph, remains below the 300-word limit, and defines nonstandard abbreviations at first use. Figure citations and captions are retained in the manuscript while the figure is supplied as a separate high-resolution file. Tables were simplified, white blood cell count units and statistical notation were standardized, and the reference list was reformatted, de-duplicated, and renumbered in order of first citation.

Location in revised files: Throughout the revised manuscript and submission package.

Comment: The data-access contact must not be an author and must be an institutional contact. Please revise the data statement and include contact information for the third-party organization.

Response: We replaced author-dependent access with an institutional controlled-access pathway. The revised statement identifies the Arab American University Institutional Review Board and Al-Makassed Islamic Charitable Society Hospital as the non-author institutional contacts, provides contact details for both institutions, explains that requests will be reviewed under applicable ethical and legal requirements and a data-use agreement, and states that the authors had no special access privileges. The underlying patient-level data are held in institutional medical and pathology records rather than a public repository; therefore, a repository dataset citation is not applicable.

Location in revised files: Main manuscript, Data availability, page 16, lines 355-363.

Comment: The ethics statement should appear only in the Methods section.

Response: The ethics statement is now located only in the Materials and methods section. It states the exact IRB reference and approval date (8 July 2025), explains that the Arab American University IRB provided ethical oversight and that Al-Makassed Hospital provided institutional permission for record access, documents the consent waiver, and describes de-identified data handling. Any duplicate ethics wording outside the Methods was removed.

Location in revised files: Main manuscript, Ethics statement, pages 7-8, lines 165-174.

Comment: If reviewer comments recommend specific publications, evaluate their relevance; citation is not mandatory unless directed by the editor.

Response: No reviewer-mandated citation was added automatically. We independently reviewed and updated the literature to support the revised knowledge-gap framing, statistical interpretation, appendicolith discussion, morphometric limitations, and reporting approach. Duplicate references were removed and bibliographic details and DOIs were checked.

Location in revised files: Main manuscript, Introduction and Discussion; revised References, pages 17-18, lines 377-425.

Academic Editor

Comment: “It is a study on a common subject... [the] study does not add anything new to the existing evidence nor identify any gaps in knowledge... [and] has a number of inconsistencies in numbers as well as interpretation of statistical tests.”

Response: We agree that the original manuscript did not adequately distinguish its contribution from the extensive existing appendicitis literature and that the numerical and interpretive inconsistencies substantially weakened the work. We therefore reframed the manuscript and performed a full source-level and statistical reconstruction rather than attempting isolated textual corrections.

The revised Introduction now explicitly states that the contribution is not the discovery of appendicolith as a new risk factor. Instead, the manuscript is positioned as a large, pathology-confirmed, source-audited Palestinian cohort that integrates routinely recorded clinical, laboratory, operative, postoperative, and gross-pathology variables; quantifies morphometric associations with confidence intervals; and reports model performance and internal validation. The title and manuscript terminology consistently use “correlates” and “associations,” and the regression is explicitly described as an association model rather than a prediction rule.

To resolve the numerical deficiencies, we reconciled the ID-linked source workbooks, reconstructed one row per unique encounter, established a transparent participant flow, regenerated every analysis, rebuilt all tables, and added variable-specific denominators. The WBC interpretation was corrected to an unexpected inverse association, CRP was no longer described as significantly different across severity categories, exact tests and adjusted post-hoc comparisons were used where appropriate, and all conclusions were rewritten to match the corrected results.

The revised Discussion and Conclusion now emphasize regional estimates, transparent negative findings, weak model discrimination, residual confounding, measurement limitations, and the need for prospective multicenter confirmation rather than overclaiming novelty or clinical utility.

Location in revised files: Main manuscript: Introduction, page 3, lines 58-71; cohort reconstruction, pages 4 and 8, lines 79-95 and 176-182; statistical methods, pages 6-7, lines 131-164; corrected Results, pages 9-12, lines 197-258; Discussion and Conclusions, pages 12-16, lines 259-354. Fig 1 and S1 Appendix added.

Reviewer #1

Comment: “This study highlights... observations and evidence already available in literature. But it could be of significance in the local/national context.”

Response: Thank you for recognizing the value of the cohort and its potential local and national relevance. We agree that the manuscript should not present established appendicitis associations as novel. The revised Introduction now explicitly acknowledges the established literature and states that the study’s contribution lies in providing a source-audited, pathology-confirmed estimate from a large Palestinian hospital cohort, together with transparent evaluation of routinely recorded gross-pathology morphometrics and comprehensive reporting of model limitations. The Discussion similarly positions the findings as contextual regional evidence and avoids claims that the appendicolith association is new.

Location in revised files: Main manuscript, Introduction, page 3, lines 58-71; Discussion, pages 12-15, lines 259-344; Conclusions, page 16, lines 345-354.

Reviewer #2

Comment: Overall assessment: the study has value because of its large cohort and pathology-confirmed outcomes, but methodological, analytical, and reporting issues require revision.

Response: We appreciate this balanced assessment. We have undertaken a comprehensive revision that addresses each methodological, analytical, and reporting concern. The cohort was reconstructed from ID-linked source data, all analyses were repeated, the manuscript was reframed as an association study, all main tables were rebuilt, a detailed S1 Appendix was added, and interpretation was made substantially more cautious.

Location in revised files: Throughout the revised manuscript and S1 Appendix.

Comment: Consistent data reporting: “N discrepancies... Provide a single clear CONSORT-style flowchart: total appendectomies identified, exclusions (with reasons and numbers), final N and per-variable denominators.”

Response: We performed a source-level reconciliation specifically to address the denominator inconsistencies. The original clinical source contained 1,287 unique Patient ID-surgery date encounters. We excluded 245 histologically normal appendices and 32 records without a classifiable histopathological severity category, yielding a final cohort of 1,010 unique encounters. The source audit identified duplicated encounter rows in the legacy analytic file and eligible source records that had not been carried into that file; the reconstructed dataset therefore uses one row per unique encounter. All analyses and tables were rerun using this cohort.

We added Fig 1, a participant-selection and cohort-reconstruction diagram, and report exact available N values in the text and tables. Table 1 and Table 2 show variable-specific denominators; Table S1 reports available and missing counts and percentages for every major variable. Percentages are explicitly calculated using available observations.

Location in revised files: Main manuscript, Participants and cohort reconstruction, page 4, lines 79-95; Results and Fig 1, page 8, lines 176-190; Tables 1-2, pages 8-9; S1 Appendix, Table S1.

Comment: Multivariable model: “Adjusted OR for WBC = 0.93... implies higher WBC is protective, yet the text states elevated WBC independently predicts complicated appendicitis. Reconcile directionality, coding and units, and correct text.”

Response: We agree. The original interpretation was incorrect. We verified the outcome coding (0=uncomplicated; 1=complicated), confirmed the WBC units, and reran the model using the reconstructed dataset. The corrected adjusted association is inverse: aOR 0.938 per 1 × 10⁹/L increase (95% CI 0.906-0.971; P<0.001). The abstract, Results, Discussion, tables, and Conclusion have all been corrected.

We do not describe WBC as protective. The revised manuscript calls the association unexpected and emphasizes that it may reflect unmeasured timing, prior treatment, surgical selection, case mix, or residual confounding. The four-category analyses also showed a non-monotonic pattern, with the significant adjusted pairwise difference occurring between gangrenous and suppurative disease.

Location in revised files: Main manuscript, Abstract, page 2, lines 18-41; primary model, page 11, lines 223-243 and Table 5; Discussion, pages 13-14, lines 276-284; Conclusions, page 16, lines 345-354; S1 Appendix, Tables S2, S3A, and S3C.

Comment: Model performance: “Present discrimination (AUC/ROC), calibration... and internal validation... if claiming predictive value.”

Response: We substantially expanded model-performance reporting and, importantly, removed predictive claims. The regression is now explicitly described as an adjusted association model rather than a clinical prediction tool. We report the likelihood-ratio chi-square, Cox-Snell and Nagelkerke R², Hosmer-Lemeshow test, AUC with 95% CI, Brier score, bootstrap optimism-corrected AUC, and bootstrap mean calibration intercept and slope. Internal validation used 300 nonparametric bootstrap resamples.

The corrected model had weak discrimination (AUC 0.594, 95% CI 0.557-0.632; optimism-corrected AUC 0.582) and low explained variation (Nagelkerke R² 0.037). These limitations are prominent in the Abstract, Results, Discussion, and Conclusion, and we explicitly state that the model should not be used as a clinical prediction tool.

Location in revised files: Main manuscript, Statistical analysis, pages 6-7, lines 141-164; Table 5 and model-performance Results, pages 11-12, lines 223-243; Discussion, pages 13-14, lines 285-292; Conclusions, page 16, lines 345-354; S1 Appendix, Table S3B.

Comment: Predictor selection, multicollinearity, functional form, and cutoffs: explain candidate-predictor selection, report VIFs, clarify how continuous variables were modeled, and consider transformations/cutoffs and sensitivity analyses.

Response: The revised Methods now state that age, sex, appendicolith, WBC, and CRP were selected a priori on the basis of clinical relevance, availability, and prior literature and were entered simultaneously; automated stepwise selection was not used. Operative approach, duration, and hospital stay were excluded from the core model because they may represent treatment selection or consequences of severity rather than baseline factors.

Continuous variables were retained in their original units to preserve information and avoid arbitrary data-derived cutoffs. Linearity in the logit was assessed using Box-Tidwell interaction terms with a Bonferroni-adjusted threshold. No core predictor crossed the adjusted threshold. Multicollinearity was assessed using tolerance and VIF; VIFs ranged from 1.004 to 1.035, indicating no meaningful multicollinearity. Because CRP showed a borderline unadjusted functional-form signal and is right-skewed, we added a sensitivity analysis with CRP on the log₂ scale, interpreted per doubling; the substantive findings were unchanged.

Location in revised files: Main manuscript, Statistical analysis, pages 6-7, lines 141-164; model diagnostics and sensitivity analyses, pages 11-12, lines 234-243; S1 Appendix, Tables S3B and S3C.

Comment: Missing important covariates and confounders: symptom duration, imaging, preoperative antibiotics, ASA class, immunosuppression, diabetes, and comorbidities should be included if available

Attachment Submitted filename: Response_to_Reviewers.docx

# Editor report: Decision Letter 1

**DOI:** 10.1371/journal.pone.0357811.r003

**Authors:** Jamshed Akhtar

15 Jul 2026

Dear Dr. Shakhshir,

Thank you for submitting your manuscript to PLOS ONE. After careful consideration, we feel that it has merit but does not fully meet PLOS ONE’s publication criteria as it currently stands. Therefore, we invite you to submit a revised version of the manuscript that addresses the points raised during the review process.

Please submit your revised manuscript by Aug 29 2026 11:59PM. If you will need more time than this to complete your revisions, please reply to this message or contact the journal office at plosone@plos.org. When you're ready to submit your revision, log on to https://www.editorialmanager.com/pone/ and select the 'Submissions Needing Revision' folder to locate your manuscript file.

- A letter that responds to each point raised by the academic editor and reviewer(s). You should upload this letter as a separate file labeled 'Response to Reviewers'.
- A marked-up copy of your manuscript that highlights changes made to the original version. You should upload this as a separate file labeled 'Revised Manuscript with Track Changes'.
- An unmarked version of your revised paper without tracked changes. You should upload this as a separate file labeled 'Manuscript'.

If you would like to make changes to your financial disclosure, please include your updated statement in your cover letter. Guidelines for resubmitting your figure files are available below the reviewer comments at the end of this letter.

If applicable, we recommend that you deposit your laboratory protocols in protocols.io to enhance the reproducibility of your results. Protocols.io assigns your protocol its own identifier (DOI) so that it can be cited independently in the future. For instructions see: https://journals.plos.org/plosone/s/submission-guidelines#loc-laboratory-protocols. Additionally, PLOS ONE offers an option for publishing peer-reviewed Lab Protocol articles, which describe protocols hosted on protocols.io. Read more information on sharing protocols at https://plos.org/protocols?utm_medium=editorial-email&utm_source=authorletters&utm_campaign=protocols.

As the corresponding author, your ORCID iD is verified in the submission system and will appear in the published article. PLOS supports the use of ORCID, and we encourage all coauthors to register for an ORCID iD and use it as well. Please encourage your coauthors to verify their ORCID iD within the submission system before final acceptance, as unverified ORCID iDs will not appear in the published article. *Only*  the individual author can complete the verification step; PLOS staff *cannot*  verify ORCID iDs on behalf of authors.

We look forward to receiving your revised manuscript.

Kind regards,

Jamshed Akhtar, MBBS, FCSP, FRCS, MHPE, FACS, M Bioethics

Academic Editor

PLOS One

Journal Requirements:

If the reviewer comments include a recommendation to cite specific previously published works, please review and evaluate these publications to determine whether they are relevant and should be cited. There is no requirement to cite these works unless the editor has indicated otherwise.

Please review your reference list to ensure that it is complete and correct. If you have cited papers that have been retracted, please include the rationale for doing so in the manuscript text, or remove these references and replace them with relevant current references. Any changes to the reference list should be mentioned in the rebuttal letter that accompanies your revised manuscript. If you need to cite a retracted article, indicate the article’s retracted status in the References list and also include a citation and full reference for the retraction notice.

Additional Editor Comments (if provided):

Abstract may be converted into a structured format keeping word count upfront. Each section should provide objective information rather than vague statements. Introduction in present format contains information like that related to the appendicolith which is not appropriate. Line 64 and onwards are out of place. This may be moved to discussion section.

In results section, readers may find it difficult to comprehend the information as for each variable denominator is different and ever time one has to refer to the text to find clarification. For example in under gender variable one record is missing and same for many other variables like operative procedure, presence of appendicolith. This may be looked into. It also raises question as to how statistical analysis was done for each category of variables.

[Note: HTML markup is below. Please do not edit.]

[NOTE: If reviewer comments were submitted as an attachment file, they will be attached to this email and accessible via the submission site. Please log into your account, locate the manuscript record, and check for the action link "View Attachments". If this link does not appear, there are no attachment files.]

To ensure your figures meet our technical requirements, please review our figure guidelines: https://journals.plos.org/plosone/s/figures

You may also use PLOS’s free figure tool, NAAS, to help you prepare publication quality figures: https://journals.plos.org/plosone/s/figures#loc-tools-for-figure-preparation.

NAAS will assess whether your figures meet our technical requirements by comparing each figure against our figure specifications.

# Author comment: Author response to Decision Letter 2

**DOI:** 10.1371/journal.pone.0357811.r004

27 Jul 2026

We thank the Academic Editor for the opportunity to submit a further revised version of our manuscript. We have addressed all comments in the current decision letter. Specifically, we converted the abstract to a structured format with objective Background, Methods, Results, and Conclusions sections; revised the Introduction by removing detailed interpretive discussion of appendicolith and moving the relevant contextual interpretation to the Discussion; and improved the presentation of variable-specific denominators and missing data by displaying the available sample size directly beside each variable in the principal tables and clarifying that group comparisons used all nonmissing observations for the relevant variable, whereas regression models used model-specific complete cases.

A detailed point-by-point response, with the locations of all revisions, has been uploaded separately as the “Response to Reviewers” file. A clean revised manuscript and a marked manuscript highlighting the changes made during this revision round have also been provided.

Attachment Submitted filename: PONE-D-26-13303R2_Response_to_Editor.docx

# Editor report: Decision Letter 2

**DOI:** 10.1371/journal.pone.0357811.r005

**Authors:** Jamshed Akhtar

23 Aug 2026

Clinical, laboratory, operative, and appendiceal morphometric correlates of histopathological appendicitis severity: a retrospective cohort study from Palestine

PONE-D-26-13303R2

Dear Dr. Shakhshir,

We’re pleased to inform you that your manuscript has been judged scientifically suitable for publication and will be formally accepted for publication once it meets all outstanding technical requirements.

Within one week, you’ll receive an e-mail detailing the required amendments. When these have been addressed, you’ll receive a formal acceptance letter and your manuscript will be scheduled for publication.

An invoice will be generated when your article is formally accepted. Please note, if your institution has a publishing partnership with PLOS and your article meets the relevant criteria, all or part of your publication costs will be covered. Please make sure your user information is up-to-date by logging into Editorial Manager at Editorial Manager® and clicking the ‘Update My Information' link at the top of the page. For questions related to billing, please contact billing support.

If your institution or institutions have a press office, please notify them about your upcoming paper to help maximize its impact. If they’ll be preparing press materials, please inform our press team as soon as possible -- no later than 48 hours after receiving the formal acceptance. Your manuscript will remain under strict press embargo until 2 pm Eastern Time on the date of publication. For more information, please contact onepress@plos.org.

Kind regards,

Jamshed Akhtar, MBBS, FCSP, FRCS, MHPE, FACS, M Bioethics

Academic Editor

PLOS One

Additional Editor Comments (optional):

Reviewers' comments:

# Editor report: Acceptance letter

**DOI:** 10.1371/journal.pone.0357811.r006

**Authors:** Jamshed Akhtar

PONE-D-26-13303R2

PLOS One

Dear Dr. Shakhshir,

I'm pleased to inform you that your manuscript has been deemed suitable for publication in PLOS One. Congratulations! Your manuscript is now being handed over to our production team.

At this stage, our production department will prepare your paper for publication. This includes ensuring the following:

* All references, tables, and figures are properly cited

* All relevant supporting information is included in the manuscript submission,

* There are no issues that prevent the paper from being properly typeset

You will receive further instructions from the production team, including instructions on how to review your proof when it is ready. Please keep in mind that we are working through a large volume of accepted articles, so please give us a few days to review your paper and let you know the next and final steps.

Lastly, if your institution or institutions have a press office, please let them know about your upcoming paper now to help maximize its impact. If they'll be preparing press materials, please inform our press team within the next 48 hours. Your manuscript will remain under strict press embargo until 2 pm Eastern Time on the date of publication. For more information, please contact onepress@plos.org.

You will receive an invoice from PLOS for your publication fee after your manuscript has reached the completed accept phase. If you receive an email requesting payment before acceptance or for any other service, this may be a phishing scheme. Learn how to identify phishing emails and protect your accounts at https://explore.plos.org/phishing.

If we can help with anything else, please email us at customercare@plos.org.

Thank you for submitting your work to PLOS One and supporting open access.

Kind regards,

PLOS One Editorial Office Staff

on behalf of

Dr. Jamshed Akhtar

Academic Editor

PLOS One
